# Phytochemical Composition, Antioxidant Activity, α-Glucosidase and Acetylcholinesterase Inhibitory Activity of Quinoa Extract and Its Fractions

**DOI:** 10.3390/molecules27082420

**Published:** 2022-04-08

**Authors:** Xi Chen, Xuemei He, Jian Sun, Zhenxing Wang

**Affiliations:** 1 Agro-Food Science and Technology Research Institute, Guangxi Academy of Agricultural Sciences, Nanning 530007, China; pangdaxi2020@163.com (X.C.); xuemeihe1981@126.com (X.H.); 2 College of Life Science, Southwest Forestry University, Kunming 650224, China; 3 Guangxi Key Laboratory of Fruits and Vegetables Storage-Processing Technology, Nanning 530007, China; 4 Key Laboratory for Forest Resources Conservation and Utilization in the Southwest Mountains of China, Ministry of Education, Southwest Forestry University, Kunming 650224, China

**Keywords:** quinoa, extract, in vitro antioxidation, α-glucosidase, acetylcholinesterase, UPLC-MS/MS

## Abstract

This study is aimed to evaluate the chemical compositions and biological activities of quinoa, a novel and excellent food crop. Quinoa extract and its fractions were prepared by ethanol extraction and liquid-liquid extraction, including ethanol crude extract, and petroleum ether, chloroform, ethyl acetate (EAF), and n-butanol and water fractions. The total phenolic and flavonoid contents, antioxidant activities, α-glucosidase and acetylcholinesterase inhibitory abilities of the extract and fractions were further determined. Based on these foundations, the chemical composition of the EAF fraction exhibiting the strongest functional activity was analyzed by ultra-performance liquid chromatography-mass spectrometry. The results showed the EAF fraction had the highest phenolic and flavonoid contents, and the highest antioxidant activities, as well as the strongest α-glucosidase and acetylcholinesterase inhibitory abilities, which is even better than the positive control. The phytochemical composition of the EAF fraction indicated that 661 and 243 metabolites were identified in positive and negative ion modes, which were classified into superclass, class and subclass levels, respectively. Phenolic acids and flavonoids were the major bioactive compounds in the EAF fraction. This study found that quinoa, especially its ethyl acetate fraction, had the potential for the development of natural antioxidants, acetylcholinesterase inhibitors, and hypoglycemic agents.

## 1. Introduction

Quinoa (*Chenopodium quinoa* Willd.) is an annual pseudo cereal that originates from the Andean region of South America. Due to its high nutritional potential and high resistance to abiotic stresses (drought, cold, and salt), quinoa is considered one of the most complete food sources for humans and has the potential to address food security under the effects of global warming and projected population growth [1,2].

With the constantly increasing demand for health, functional studies of quinoa have gained substantial attention in recent years. A number of studies have reported the various biological activities of quinoa, such as antioxidant, anticancer, anti-inflammatory, antibacterial, antidiabetic, and immune regulation, which are attributed to its abundant bioactive ingredients, including flavonoids, phenolic acids, betalains, polysaccharides, and saponins, and these bioactive components in quinoa are considered to be excellent natural antioxidants [3,4,5,6,7,8]. It is well known that supplementation of antioxidants is vital to combating oxidative stress, which is associated with many diseases, including Type 2 diabetes (T2D), Alzheimer’s disease (AD), cancer, cardiovascular diseases, and neurological disorders [9,10]. When compared to synthetic antioxidants, natural antioxidants have attracted substantial attention by virtue of their advantages (safety, high efficiency, and without side effects). Based on those excellent function properties, it is of great interest to extract the active ingredients in quinoa and explore their functions.

In terms of the existing research on quinoa, most research has focused on phytochemical screening and analysis, the function of the active ingredients or fractions, and the impact of cultivation modes and processing parameters on these active components’ content [7,11,12,13]. In general, different extraction methods have significant effects on the chemical and functional properties of the natural active ingredients, especially solvent and other conditions. To the best of the authors’ knowledge, research about the effect of extraction solvents on the function activities and the functional components from different parts of quinoa is still scarce.

Hence, in the present study, quinoa was sequentially extracted using several solvents, then the in vitro antioxidant, anti-diabetic, acetylcholine esterase inhibitory of different polar solvents extracts were investigated. Finally, major components of the most active fraction were identified using ultra-performance liquid chromatography-mass spectrometry (UPLC-MS/MS). Considering the worldwide diffusion of quinoa cultivation, especially the increasing planted area in China, such as Shangri La, Lijiang, and Diqing of Yunnan Province, this study could provide a basis for the further development and utilization of quinoa and is helpful for human healthy lives.

## 2. Results and Discussion

### 2.1. Total Phenolics and Flavonoids Content

Polyphenols are one of the most abundant types of plant secondary metabolites, and flavonoids and phenolic acids are the two most common polyphenol compounds, which exhibit excellent biological activities, such as antioxidant and antidiabetic activities. [14,15]. The results of total phenolics content (TPC) and total flavonoids content (TFC) of different fractions were presented in Table 1. The ethyl acetate fraction (EAF) showed the highest TPC (345.57 mg/g) and TFC (279.36 mg/g), followed by crude extract (CE), n-butanol fraction (NF), chloroform fraction (CF), water residual (WR), and petroleum ether fraction (PEF). EAF was 13.95 and 10.29-fold of that in PEF, respectively. This was in agreement with the previous finding that the ethyl acetate fraction exhibited the highest TPC and TFC values; the TPC and TFC of petroleum ether extract, were the lowest [16].

### 2.2. In Vitro Antioxidant Activities

Based on the free radical scavenging principle, the antioxidant activity of quinoa was measured. Considering different methods have different mechanisms and limitations, which may lead to different results, four assays (2,2-diphenyl-1-picrylhydrazyl (DPPH) assay, 2,2′-azino-bis (3-ethylbenzothiazoline-6-sulfonic acid) (ABTS) assay, ferric reducing antioxidant power (FRAP) assay, and oxygen radical absorbance capacity (ORAC) assay were used in this study, rather than depending on a single assay.

#### 2.2.1. DPPH Radical Scavenging Activity

**Figure 1:**
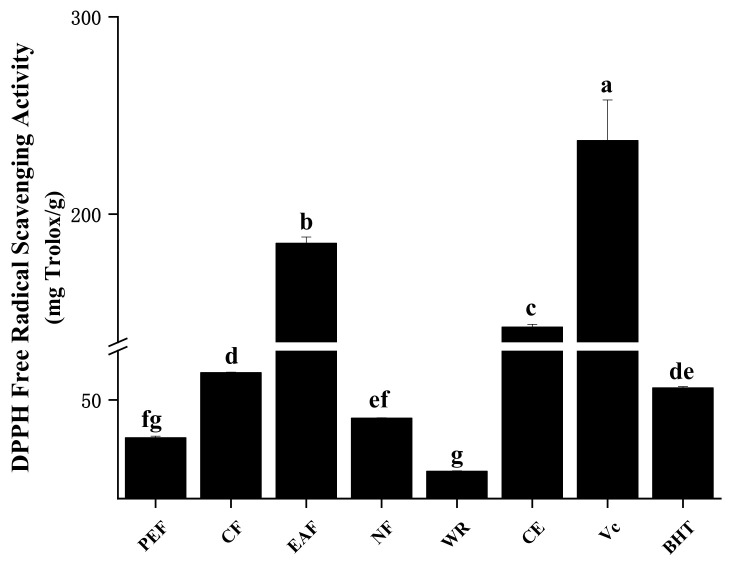
The DPPH radical scavenging ability of samples. The different letters in the column represented significantly difference (*p* < 0.05).

DPPH radical scavenging ability is a commonly used method to appraise the antioxidant activity of natural compounds. During the reaction, the nitrogen-free radical is scavenged by antioxidative compounds, and the violet color became lighter, which can be quantified spectroscopically at a wavelength of 515–528 nm [17]. As displayed in Figure 1, EAF had the strongest DPPH radical scavenging activities (185.09 ± 3.30 mg Trolox/g), followed by CE (142.66 ± 1.45 mg Trolox/g), CF (63.79 ± 0.29 mg Trolox/g), NF (40.76 ± 0.19 mg Trolox/g), PEF (30.82 ± 0.82 mg Trolox/g), and WR (13.78 ± 0.34 mg Trolox/g). Although the activity of all fractions was weaker than the positive control Vc (237.24 ± 20.60 mg Trolox/g), EAF and CE were stronger than other commonly used commercial antioxidants BHT (56.08 ± 0.69 mg Trolox/g). The correlation analysis (Table 2) showed that DPPH free radical scavenging ability was extremely significantly associated with TPC and TFC (r = 0.971 and 0.952, *p* < 0.01). The results suggested that polyphenolic compounds, especially flavonoids, are the main components of DPPH radical scavenging ability.

#### 2.2.2. ABTS Radical Scavenging Activity

**Figure 2:**
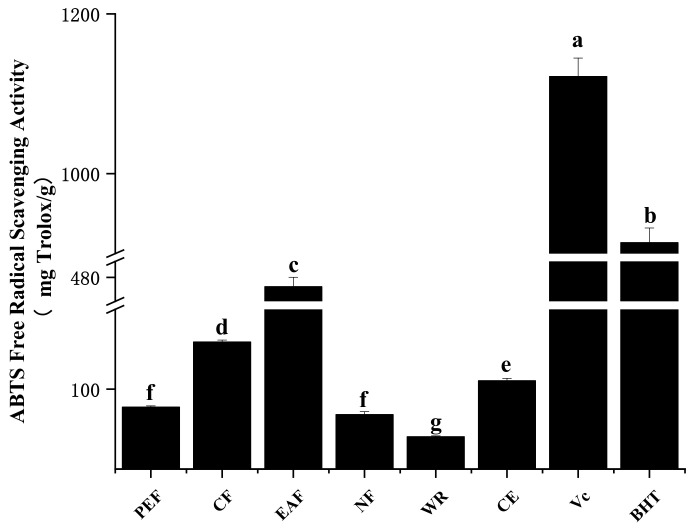
The ABTS radical scavenging ability of samples. The different letters in the column represented significantly difference (*p* < 0.05).

In contrast to DPPH radical scavenging ability, ABTS radical scavenging activity is more reactive and involves an electron transfer process [18]. From Figure 2, the trend for ABTS radical scavenging activity was similar to DPPH radical scavenging activity. The EAF fraction gave the strongest ABTS radical scavenging activity (468.34 ± 11.89 mg Trolox/g), followed by CF (159.04 ± 2.48 mg Trolox/g), CE (110.48 ± 3.12 mg Trolox/g), PEF (77.54 ± 1.62 mg Trolox/g), NF (68.16 ± 3.63 mg Trolox/g), WR (40.46 ± 1.13 mg Trolox/g); EAF which was 11.7 times higher than WR. Although they were all significantly lower than Vc (1121.49 ± 23.16 mg Trolox/g) and BHT (913.46 ± 18.48 mg Trolox/g). Similarly, the correlation of ABTS radical scavenging ability with TPC and TFC remained significant (r = 0.848 and 0.895, *p* < 0.01), which indicated that phenolic acids and flavonoids were excellent DPPH radical scavengers. In addition, a significant relationship between ABTS radical scavenging ability and DPPH radical scavenging ability was observed (r = 0.820, *p* < 0.01). This result indicated that the critical active components in quinoa have similar effects for these two indices.

#### 2.2.3. Ferric Reducing Antioxidant Power

**Figure 3:**
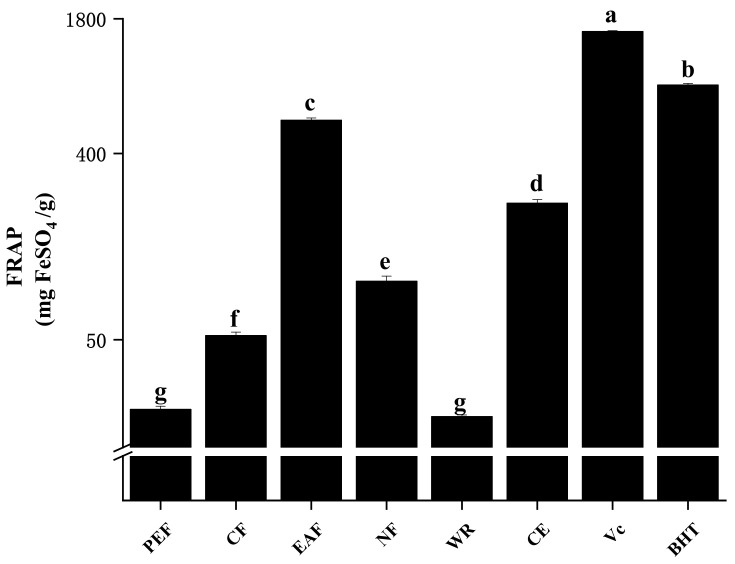
The ferric reducing antioxidant power of samples. The different letters in the column represented significantly difference (*p* < 0.05).

The ferric reducing antioxidant power (FRAP) assay is a widely used method to estimate the reducing potential of antioxidants. During the reaction, samples were reacted with a ferric tripyridyltriazine (Fe^3+^-TPTZ) complex and produced a blue-colored ferrous tripyridyltriazine (Fe^2+^-TPTZ); the antioxidant capacity was evaluated by the degree of color change [19]. According to Figure 3, although these antioxidant capacities lower than the control Vc (1560.86 ± 15.69 mg of FeSO_4_/g) and BHT (857.59 ± 17.22 mg of FeSO_4_/g), EAF still showed the highest FRAP value (579.84 ± 14.41 mg FeSO_4_/g), followed by CE (229.33 ± 9.92 mg FeSO_4_/g), NF (95.96 ± 5.72 mg FeSO_4_/g), CF (52.34 ± 2.04 mg FeSO_4_/g), PEF (22.94 ± 0.85 mg FeSO_4_/g), and WR (21.15 ± 0.44 mg FeSO_4_/g). The high significant correlations among FRAP with TPC, TFC, DPPH radical scavenging ability, and ABTS radical scavenging ability (r = 0.923–0.981, *p* < 0.01) indicated that FRAP could reflect the antioxidant capacity more comprehensively, shown in Table 2.

#### 2.2.4. Oxygen Radical Absorbance Capacity

**Figure 4:**
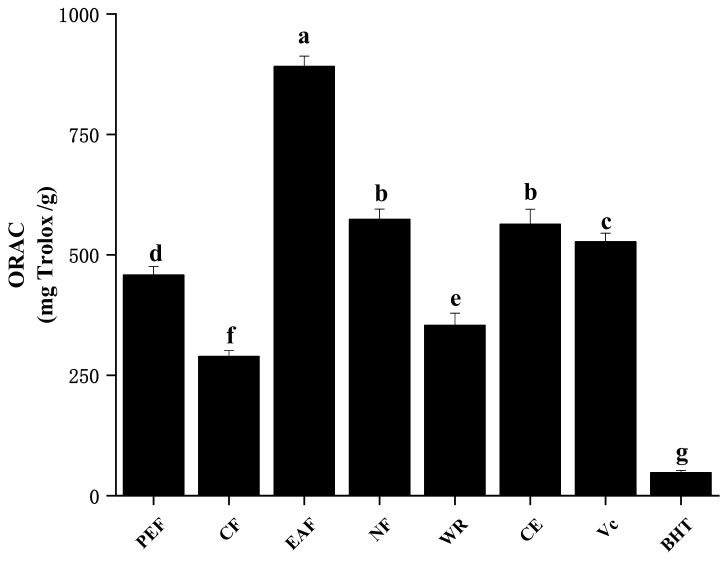
The oxygen radical absorbance capacity of samples. The different letters in the column represented significantly difference (*p* < 0.05).

The oxygen radical absorbance capacity (ORAC) assay measured the antioxidant-mediated inhibition of peroxyl radical-induced oxidation and thus, reflected classical radical chain-breaking antioxidant activity by hydrogen atom transfer, while FRAP, ABTS and DPPH measure the electron transfer [20,21]. As shown in Figure 4, the EAF fraction presented the highest ORAC values (891.13 ± 21.45 mg Trolox/g), followed by NF (573.78 ± 21.13 mg Trolox/g) and CE (563.59 ± 31.17 mg Trolox/g); they exhibited stronger antioxidant capacity when compared the positive control Vc (527.06 ± 17.81 mg Trolox/g). It was worth stating that all the fractions were outperformed by another commonly used antioxidant BHT (47.88 ± 4.77 mg Trolox/g). These results were similar to those reported elsewhere [5,12]. The high and significant correlation between ORAC and TPC, TFC, and other antioxidant indicators (r = 0.778–0.917, *p* < 0.01) revealed that phenolic and flavonoids in quinoa played important roles in absorbing oxygen-free radicals, and ORAC was closer and similar to FRAP.

### 2.3. α-Glucosidase Inhibitory Activity

**Figure 5:**
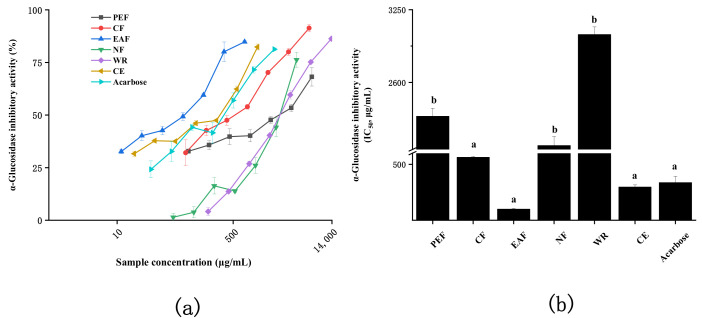
α-Glucosidase inhibitory activity of bioactive fractions from quinoa. (**a**) The inhibition rate of the α-glucosidase with different concentrations. (**b**) The IC_50_ values of different samples toward α-glucosidase inhibitory activity. The different letters in the column represented significantly difference (*p* < 0.05).

T2D has been a primary metabolic disease worldwide. A primary therapy is to prevent postprandial hyperglycemia by suppressing hydrolysis of carbohydrates, and α-glucosidase is an important target [22]. The α-glucosidase inhibitory effect of quinoa is shown in Figure 5. All extracts significantly inhibited the α-glucosidase in a concentration-dependent manner (Figure 5a), wherein EAF showed the best results (IC_50_ value was 99.66 ± 6.00 µg/mL), followed by CE (297.08 ± 18.54 µg/mL), CF (562.44 ± 7.88 µg/mL), NF (2034.96 ± 80.65 µg/mL), PEF (2296.60 ± 72.14 µg/mL), and WR (3027.44 ± 68.69 µg/mL), which was similar to the results of Tang [23]. Surprisingly, EAF and CE performed significantly better than acarbose (336.25 ± 56.88 µg/mL), a commonly used commercial hypoglycemic agent (Figure 5b). Correlation analysis showed that the α-glucosidase inhibitory ability of quinoa was largely attributed to the presence of phenolics and flavonoids (Table 2), which were also the major contributors to the antioxidant capacity. This was also evidenced by the high correlation between α-glucosidase inhibitory ability and antioxidant capacity. Oxidative stress, in particular reactive oxygen species (ROS), play an important role in the induction of diabetes and its associated complications, so dietary antioxidants play a vital role in managing oxidative stress and diabetes [24]. The results indicated the great potential of quinoa as a natural hypoglycemic agent.

### 2.4. Acetylcholinesterase Inhibitory Activity

**Figure 6:**
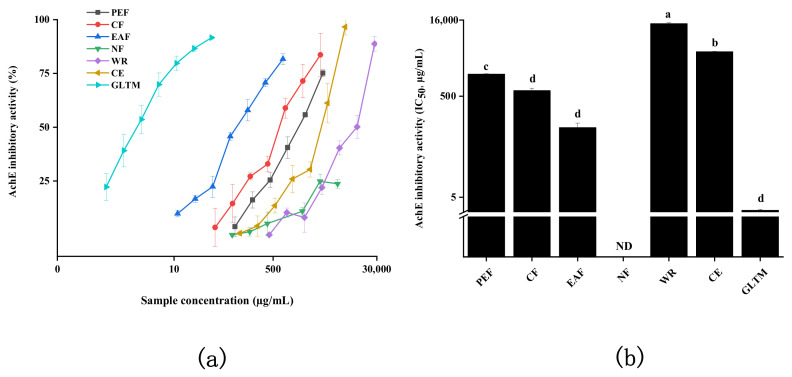
AChE inhibition of bioactive fractions from quinoa. (**a**) The inhibition rate of the AChE with different concentrations. (**b**) The IC_50_ values of different samples toward AChE inhibitory activity. The different letters in the column represented significantly difference (*p* < 0.05).

Acetylcholinesterase (AChE) is one of the major targets for the treatment of AD, and AChE inhibitors are thought to be clinically validated treatments [25]. Like diabetes, the association between AChE inhibition and increased oxidative stress was also demonstrated [26]. As presented in Figure 6, all the extracts have the ability to inhibit AChE except NF. Although these inhibition activities are lower than the standard drug GLTM (IC_50_ value was 2.76 ± 0.15 µg/mL), EAF still has the strongest inhibition activity (IC_50_ value was 118.91 ± 28.06 µg/mL), which was 6, 12, 32, and 115 times higher than CF, PEF, CE, and WR, respectively. Correlation analysis showed that the AChE inhibitory activity was not significantly related to TPC, TFC, and different antioxidant capacities except ABTS radical scavenging ability. This suggested that AChE inhibitory activity may be the combined result of multiple ingredients, not only caused by these compounds with antioxidant effects, such as phenolics and flavonoids. Taking into consideration that quinoa could be ingested safely and in large amounts as a food, it is of great significance to explore the potential of quinoa as a novel dietary supplement-drug for AD.

### 2.5. Ultra-Performance Liquid Chromatography-Mass Spectrometry Analysis

**Figure 7:**
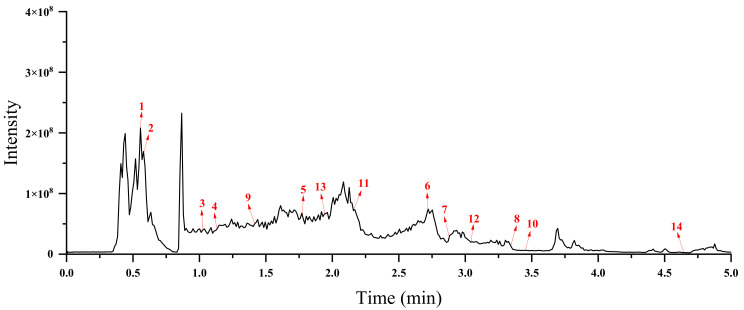
Total ion current chromatogram (TIC) of EAF fraction of crude extract from quinoa at negative ion mode.

Based on the results described above, the EAF fraction demonstrated the highest TPC and TFC, the strongest antioxidant activity, α-glucosidase inhibitory ability, and AChE inhibitory activity. Therefore, the major chemical components of the EAF fraction were investigated by ultra-performance liquid chromatography-mass spectrometry (UPLC-MS/MS), and the total ion chromatograms (TIC) in negative ion modes were presented in Figure 7. The metabolites were identified by comparing their accurate mass (error < 10 ppm), retention time, fragmentation pattern, and collision energy with a database of metabolite reference standards (In-house database by Shanghai Applied Protein Technology Co., Ltd, Shanghai, China.). According to the confidence levels of compound annotations discussed by the compound identification workgroup of the Metabolomics Society at the 2017 annual meeting of the Metabolomics Society, all the confidences of the metabolites identified in this study were level 2 or higher [27].

**Figure 8:**
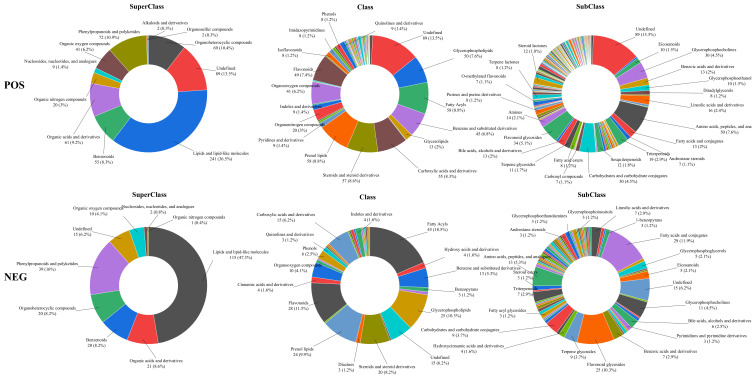
The schematic diagram of the different classifications of the metabolites of EAF fraction of crude extract from quinoa.

**Figure 9:**
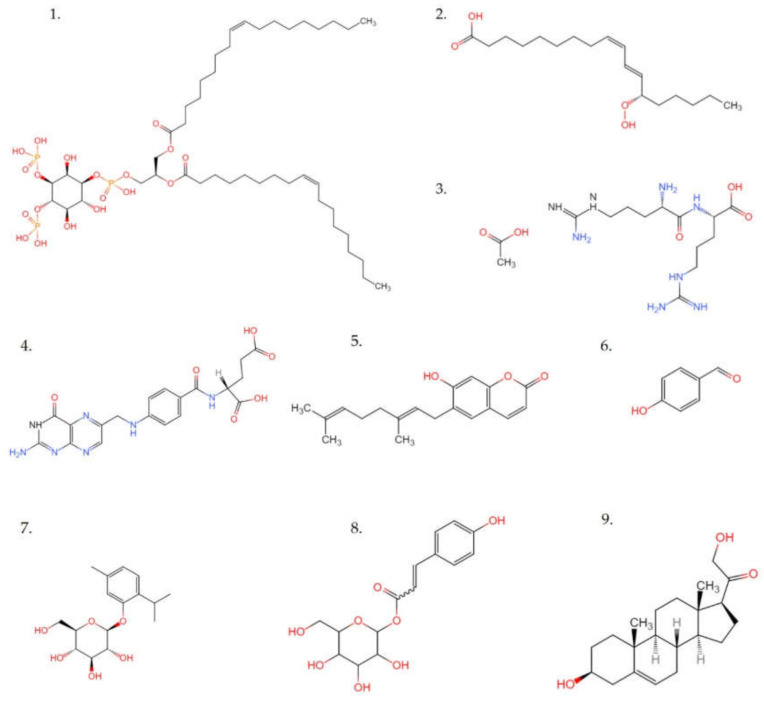
Chemical structures of representative compounds identified in the EAF fraction of crude extract from quinoa by UPLC-MS/MS. **1**: 1,2-dioleoyl-sn-glycero-3-phospho-(1′-myo-inositol-3′,4′-bisphosphate); **2**: 13s-hydroperoxy-9z,11e-octadecadienoic acid; **3**: Arg-Arg; **4**: Folate; **5**: Ostruthin; **6**: 4-hydroxybenzaldehyde; **7**: Thymol-beta-d-glucoside; **8**: Coumaroyl hexoside; **9**: 21-Hydroxypregnenolone.

In positive ion mode, 661 metabolites were identified and included 243 metabolites in negative ion, which were presented in the supporting files (Supplementary Materials: Table S1). The classifications of these metabolites were given in Figure 8, and only classification proportions greater than 1% were shown at the class and subclass levels. The different colors in each pie chart represented different classifications, and the area represented the relative proportion of metabolites in the classification. As exhibited in Figure 8, the predominant superclass metabolites were lipids and lipid-like molecules, phenylpropanoids and polyketides, organic acids and derivatives, benzenoids, and organoheterocyclic compounds. At the class level, they were mainly fatty acyls, prenol lipids, steroids and steroid derivatives, carboxylic acids and derivatives, glycerophospholipids, as well as flavonoids. While at the subclass level, they were amino acids, peptides, analogs, flavonoid glycosides, glycerophosphocholines, carbohydrates, carbohydrate conjugates, and triterpenoids. The representative compounds were shown in Figure 9. Thus, these metabolites might be the basis for the functional activity of the EAF.

**Figure 10:**
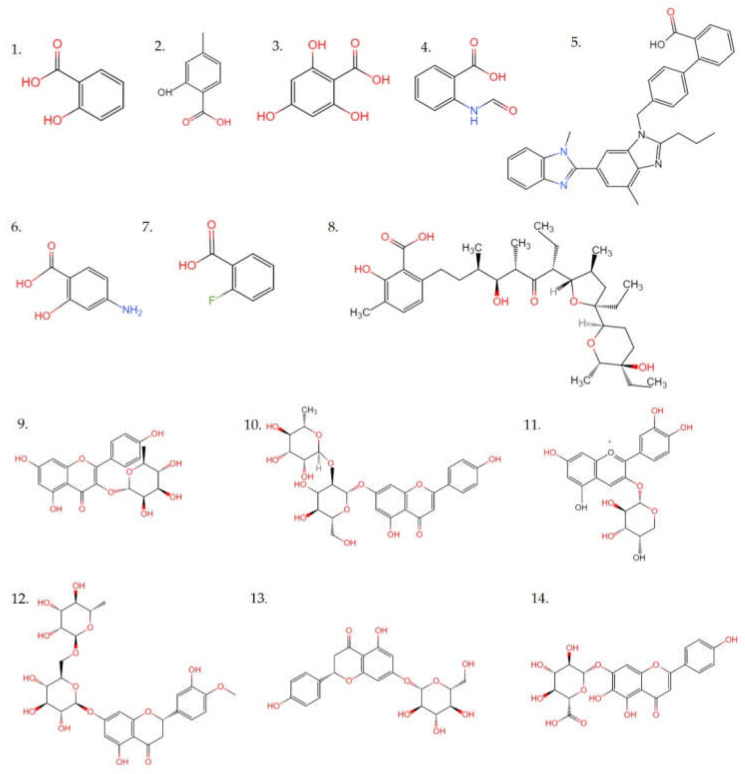
Chemical structures of 14 phenolic acids identified in the fraction of crude extract from quinoa by UPLC-MS/MS. **1**: Salicylic acid; **2**: 2-hydroxy-4-methylbenzoic acid; **3**: Phloroglucinolcar-boxylic acid; **4**: Formylanthranilic acid; **5**: Telmisartan; **6**: 4-Aminosalicylic acid; **7**: 2-Fluorobenzoic acid; **8**: Lasalocid; **9**: Afzelin; **10**: Apigenin-7-O-neohesperidoside; **11**: Cyanidin-3-O-alpha-arabinoside; **12**: Hesperidin; **13**: Naringenin-7-O-glucoside; **14**: Scutellarin.

Although phenols (including flavonoids) metabolites were not the most identified in mass spectrometry, considering the high TPC and TFC in EAF, and the important role of phenols in these functional activities, such as antioxidant activity and α-glucosidase inhibitory ability, the major phenolic acid and flavonoids in EAF fraction were further characterized. A total of eight phenolic acids were identified, including 2-hydroxy-4-methylbenzoic acid, 4-aminosalicylic acid, benzoic acid, 2-fluoro- formylanthranilic acid, phloroglucinolcarboxylic acid, lasalocid, salicylic acid and telmisartan. Six flavonoids were identified in the EAF fraction, which contained afzelin, apigenin-7-O-neohesperidoside, cyanidin-3-O-alpha-arabinoside, hesperidin, naringenin-7-O-glucoside and scutellarin. The detailed information on these compounds was shown in Figure 10 and listed in Table 3. In the previous study by Lin et al. [28], 29 phenolic acid analogs were identified from quinoa. This probably was due to EAF being the secondary organic extract generated by liquid–liquid extraction in this study, while it was crude extract in Lin’s study. Among these compounds, NO. 2, 4, 6, and 7 were benzoic acid analogs, which had been documented to have antioxidant and antibacterial functions [28]. The other substances also have many important biological activities. Salicylic acid played a central role in plant immunity, which has the function of indirectly controlling both cell death and cell survival [29]. Phloroglucinolcar-boxylic acid could inhibit Cyclin Dependent Kinase (CDK) activity and cancer cell proliferation [30]. Telmisartan was safe and effective in the treatment of arterial hypertension [31]. Lasalocid, a polyether carboxylic acid ionophore, was effective as a coccidiostat in poultry and was also used extensively for improving feed efficiency in ruminants [32]. These biological activities would provide greater possibility for deep processing and utilization of quinoa. A previous study has reported that the antioxidant activity, and α-glucosidase and pancreatic lipase inhibitory activities of plant extracts were significantly positively correlated with their phenolic acids content [23]. Phenolics have special and highly stable chemical structures, which resulted in their well-known functional activity, including antioxidant, anti-inflammatory, anticancer, antidiabetic, anti-obesity effects [33,34]. Therefore, it is important to study the phenolics in quinoa.

## 3. Materials and Methods

### 3.1. Materials and Reagents

α-Glucosidase, acarbose, 4-methyl-umbrella-ketone-α-d-glucopyranoside (4-MUG), acetylcholinesterase (AChE), galanthamine (GLTM), ammonium acetate, acetylthiocholine iodide (ATCI), 5,5′-dithiobis (2-nitrobenzoic acid) (DNTB) were purchased from Sigma- Aldrich (St. Louis, MO, USA). Chromatographic acetonitrile was purchased from Merck (Darmstadt, Germany). 2,2′-diazo-bis (3-ethylbenzothiazoline-6-sulfonic acid) (ABTS), 2,2-diphenyl-1-picrylhydrazyl (DPPH), 2,4,6-tripyridyl triazine (TPTZ), 2,2′-azo-bis-(2-amidopropane) dihydrochloride (AAPH), sodium fluorescein (FL), water-soluble vitamin E (Trolox), vitamin C (Vc), 2,6-Di-tert-butyl-4-methylphenol (BHT), and Folin-phenol reagent were purchased from Solarbio (Beijing, China). Other analytical grade chemicals, including methanol, petroleum ether, ethyl acetate, n-butanol and ethanol were purchased from Xilong Chemical Co., Ltd. (Shantou, China).

### 3.2. Sample Preparation

Quinoa was obtained from Shangri La, Di Qing Tibetan Autonomous Prefecture, Yunnan Province. Quinoa (200 g) was first dried to constant weight, crushed and sifted through a 40-mesh sieve. The powder of quinoa was extracted with a 70% ethanol solution (solid-liquid ratio of 1:20, ultrasonic power 500 W) by an ultrasonic cleaner (SG5200HDT, Shanghai guante Ultrasonic Instrument Co., Ltd., Shanghai, China) at 50 °C for 1 h. The extracted solution was collected, and the residues were re-extracted under the same conditions, and the combined solution was concentrated under a vacuum at 50 °C using a rotary evaporator (Shanghai Yarong biochemical instrument factory, Shanghai, China) to yield the ethanol crude extract of quinoa. The crude extract was dissolved with distilled water and extracted successively with petroleum ether, chloroform, ethyl acetate and n-butanol (volume ratio of 1:1) three times. The same fractions were combined and concentrated to obtain petroleum ether, chloroform, ethyl acetate, n-butanol and water fractions.

### 3.3. Total Phenolics Content (TPC)

TPC was measured based on the Folin-phenol method reported by Tang et al. [35] with a few modifications. The tested sample (50 µL) was mixed with Folin-phenol reagent (125 µL) and 7.5% Na_2_CO_3_ (100 µL) on a 96-well microplate. The absorbance of the mixture was measured at 765 nm using a multimode reader (Synergy H1, Bio Tek, Winooski, VT, USA), after incubating for 30 min in the dark. The tested sample was diluted with 70% ethanol solution (the same below) to a suitable concentration. Gallic acid was used as an external standard. The TPC of samples was represented as milligrams of gallic acid equivalents per gram of quinoa dried sample (mg of GAE/g).

### 3.4. Total Flavonoids Content (TFC)

TFC was determined by a modified sodium nitrite-aluminum nitrate colorimetric assay method according to Tang et al. [35]. Rutin solution or sample with a certain concentration (40 µL) was reacted with 3% NaNO_2_ (20 µL) in a 96-well microplate for 6 min, and then added 6% Al (NO_3_)_3_ (20 µL) incubating for 6 min. Finally, the absorbances at 510 nm were detected after the addition of 4% NaOH (140 µL) and 70% ethanol (60 µL) for incubating for 15 min. The TFC was calculated as the method of TPC and expressed as milligrams of rutin equivalents per gram of quinoa dried sample (mg of RE/g).

### 3.5. Antioxidant Activities

#### 3.5.1. DPPH Radical Scavenging Activity

DPPH radical scavenging capacity was performed following the protocol reported by Tang et al. [36]. The sample with an appropriate concentration (100 µL) was mixed with 0.15 mmol/L DPPH (100 µL) in a 96-well microplate, and the mixture was reacted in the dark for 30 min, and then the absorbances (A_S_) at 517 nm were measured. The control group A_c_ (70% ethanol was used instead of the sample) and blank group A_b_ (70% ethanol was used instead of the DPPH solution) were set up simultaneously, and Vc and BHT were assigned as the positive control. The DPPH scavenging percentage was calculated as follows. Trolox was used as a standard and the DPPH free radical scavenging capacity was expressed as Trolox equivalents (mg of Trolox/g).

$$R\;=\;\frac{A_{c}\;-\;(A_{s}\;-\;A_{b})}{A_{c}}\;\times \;100\%$$

where R is scavenging effect (%); A_s_ is the absorbance value of sample; A_c_ is the absorbance value of control group, using the 70% ethanol instead of sample; A_b_ is the absorbance value of the blank group, using the 70% ethanol instead of the DPPH solution.

#### 3.5.2. ABTS Radical Scavenging Activity

ABTS radical scavenging capacity was carried out according to Guedes et al. [37]. The sample with an appropriate concentration (50 µL) was added with 7 mmol/L prepared ABTS (200 µL) on a 96-well microplate, and the mixture was left standing for 5 min in the dark, and then the absorbances were measured at 734 nm. The calculation method of ABTS scavenging percentage was the same as Section 3.5.1.

#### 3.5.3. Ferric Reducing Antioxidant Power (FRAP)

FRAP was determined on the basis of the method reported by Escribano et al. [5]. The sample with an appropriate concentration (30 µL) was mixed with 240 µL FRAP solution (contained 300 mmol/L pH 3.6 sodium acetate buffer, 10 mmol/L TPTZ and 20 mmol/L FeCl_3_ with a volume ratio of 10:1:1). The mixture was incubated for 10 min in the dark at 37 °C, and then the absorbance at 593 nm was measured. The calculation method of FRAP was the same as Section 3.5.1. Results were represented as milligrams of FeSO_4_ equivalents per gram of quinoa dried sample (mg of FeSO_4_/g).

#### 3.5.4. Oxygen Radical Absorbance Capacity (ORAC)

The ORAC assay was performed as described by Hernández-Ledesma et al. [38]. The ORAC was determined by a multimode reader (Synergy H1, Bio Tek, Winooski, VT, USA), using a Trolox-AUC calibration curve and represented as Trolox equivalents (mg of Trolox/g).

### 3.6. Enzyme Inhibitory Ability

#### 3.6.1. Inhibition activity of α-Glucosidase

The α-glucosidase inhibition activity was measured as previously described by Liao et al. [39]. α-Glucosidase and 4-MUG solution were prepared by potassium phosphate buffer (pH 6.8). The sample with an appropriate concentration (50 µL) was mixed with 20 µL α-glucosidase solution (0.175 U/mL) and 50 μL 4-MUG (0.84 μmol), and the mixture was incubated at 37 °C for 20 min in darkness. Then, the reaction was terminated by adding 100 µL sodium glycine solution (100 mmol, pH 10.6) and oscillating for 30 s. The excitation and emission wavelength were set as 355 nm and 460 nm, and then the fluorescence was monitored by a multimode reader (Synergy H1, Bio Tek, Winooski, VT, USA). Acarbose was assigned as a positive control, and the inhibition rate of α-glucosidase was calculated as follows:

$$R\;=\;\frac{(A_{c}\;-\;A_{b})\;-\;\left(A_{s}\;-\;A_{b}\right)}{A_{c}\;-\;A_{b}}\;\times \;100\%$$

where R is inhibition rate (%); A_s_ is the fluorescence of quinoa sample; A_c_ is the fluorescence of control, using the 70% ethanol instead of sample; A_b_ is the fluorescence of blank group, using the potassium phosphate buffer instead of the α-glucosidase solution. The sample concentration required to produce 50% inhibition was defined as the IC_50_ value.

#### 3.6.2. Inhibition Activity of Acetylcholinesterase

AChE inhibition activity was determined based on the method reported by Lee et al. [40]; 50 µL of sample solution, 15 µL ATCI solution (15 mM, water-solution) and 75 μL DTNB (3 μmol, pH 8.0 sodium phosphate buffer) were added into 96-well plate. After a 10 min incubation at 30 °C, the 20 μL AChE solution (0.1 U/mL, pH 8.0 sodium phosphate buffer) was added. At last, the reaction was terminated by adding 50 µL of sodium phosphate buffer. The absorbance was monitored every 1 min, five times (the plates were shaken for 10 s before each measurement) at 405 nm by a multimode reader (Synergy H1, Bio Tek, Winooski, VT, USA). The 70% ethanol was used as a control group while the sodium phosphate buffer was chosen as a blank group. GLTM was assigned as a positive control, and the inhibition rate of AChE was calculated as Section 3.6.1.

### 3.7. UPLC-MS/MS Analysis

The UPLC-MS/MS analysis was performed according to the previously described method [41]. The samples were separated on a 1290 UHPLC system (Agilent Technologies Inc., Palo Alto, CA, USA) equipped with an ACQUITY UPLC BEH Amide column (2.1 mm × 100 mm, 1.7 μm, Waters, Milford, MA, USA). The column temperature was 25 °C. Flow rate: 0.5 mL/min, injection volume 2 μL. The mobile phase consisted of water, ammonium acetate (25 mM) and ammonia (25 mM) (A), and acetonitrile (B). The gradient elution procedure was listed in Table 4. During the whole analysis process, the samples were placed in a 4 °C automatic sampler.

The mass spectrometer (Triple TOF 6600, AB Sciex, Framingham, MA, USA) was used to collect the primary and secondary spectra of samples. The ESI source conditions were as follows: ion source Gas1 (Gas1), 60; ion source Gas2 (Gas2), 60; curtain Gas (CUR), 30; source temperature, 600 °C; ionsapary voltage floating (ISVF) ± 5500 V (positive and negative modes); TOF MS scan *m*/*z* range, 60–1000 Da; product ion scan *m*/*z* range, 25–1000 Da; TOF MS scan accumulation time, 0.20 s/spectra; product ion scan accumulation time, 0.05 s/spectra; The secondary mass spectrum was obtained by information dependent acquisition (IDA), and the high sensitivity mode was adopted. The declustering potential (DP) was ± 60 V (positive and negative modes) and collaboration energy was 35 ± 15 EV. The IDA settings were as follows: exclude isotopes within 4 Da; candidate ions to monitor per cycle, 10.

By matching with the retention time, molecular weight (molecular weight error < 25 ppm), secondary fragmentation spectrum, collision energy and other information of metabolites in the local database, the structure of metabolites in samples were identified, and part of the results were manually checked.

### 3.8. Statistical Analysis

All experiments were repeated three times and the results were represented as the mean ± standard deviation. All analyses were performed using SPSS software (IBM Co. Chicago, IL, USA, version 25.0) and considered significant for *p* < 0.05. Curve fitting was performed and IC_50_ values were calculated with Origin software (OriginLab Co., Northampton, MA, USA, version 2018). Different letters on the column showed significant differences.

## 4. Conclusions

In this study, quinoa was extracted and then further partitioned using various solvents, such as petroleum ether, chloroform, ethyl acetate, n-butanol and water fractions, respectively. The total phenolics and flavonoids contents, antioxidant activities, α-glucosidase and AChE inhibitory abilities of quinoa extract and its fractions were evaluated. The results showed that quinoa extract and its fractions had high active ingredient content and excellent functional activity compared to other food crops, such as wheat and barley. Of these, EAF had the highest TPC and TFC, and the highest antioxidant activity, including DPPH and ABTS radical scavenging abilities, FRAP, and ORAC. In addition, EAF also showed the strongest α-glucosidase and AChE inhibitory abilities, which are even better than acarbose, a commonly oral anti-diabetic drug. The material basis of the EAF was confirmed by UPLC-MS/MS, phenolic acids and flavonoids were the main active compounds in the EAF fraction. These results indicated that quinoa, especially its EAF fraction, had the potential for the development of natural antioxidants, AChE inhibitors, and hypoglycemic drugs. This study could provide a scientific basis for the utilization of active components in quinoa.

## Figures and Tables

**Table 1:** The TPC and TFC of quinoa extracts.

Samples	TPC	TFC
PEF	16.03 ± 2.49 ^a^	14.31 ± 2.17 ^a^
CF	86.74 ± 6.55 ^b^	68.94 ± 4.86 ^b^
EAF	345.57 ± 6.80 ^e^	279.36 ± 6.47 ^e^
NF	102.78 ± 8.33 ^c^	88.19 ± 7.43 ^c^
WR	27.10 ± 2.20 ^a^	18.02 ± 1.83 ^a^
CE	223.66 ± 11.35 ^d^	147.26 ± 8.00 ^d^

TPC = total phenolic content (mg GAE/g); TFC = total flavonoid content (mg RE/g); PEF = petroleum ether fraction; CF = chloroform fraction; EAF = ethyl acetate fraction; NF = n-butanol fraction; CE = crude extract and WR = water residual. The means with different lowercase letters in the same column are significantly different (*p* < 0.05).

**Table 2:** The correlational analysis of each index of samples.

	TPC	TFC	DPPH	ABTS	FRAP	ORAC	α-Glucosidase	AChE
TPC	1							
TFC	0.987 **	1						
DPPH	0.971 **	0.952 **	1	-	-	-	-	-
ABTS	0.848 **	0.895 **	0.820 **	1	-	-	-	-
FRAP	0.963 **	0.981 **	0.923 **	0.929 **	1	-	-	-
ORAC	0.855 **	0.883 **	0.793 **	0.778 **	0.917 **	1	-	-
α-glucosidase	−0.814 **	−0.773 **	−0.860 **	−0.672 **	−0.687 *	−0.467	1	-
AChE	−0.439	−0.466	−0.526	−0.547 *	−0.419	−0.398	0.572 *	1

TPC = total phenolics content; TFC = total flavonoids content; DPPH = DPPH scavenging activity; ABTS = ABTS·+ scavenging ability; FRAP = ferric reducing antioxidant power; ORAC = oxygen radical absorbance capacity; α-glucosidase = the IC_50_ value for α-glucosidase inhibitory activity; AChE = the IC_50_ value for acetylcholinesterase inhibitory activity. * indicates significant correlation, *p* < 0.05; ** indicates extremely significant correlation, *p* < 0.01.

**Table 3:** Identification of phenolic acids and flavonoids in the EAF fraction of crude extract from quinoa by UPLC-MS/MS.

No.	Name of Compounds	RT(S)	Molecular Formula	Molecular Weight	MS/MS	Type
1	Salicylic acid	33.017	C_7_H_6_O_3_	138.12	93.0124, 75.0041	Phenolic acids
2	2-hydroxy-4-methylbenzoic acid	34.092	C_8_H_7_O_3_	151.14	107.0253, 92.0283	Phenolic acids
3	Phloroglucinolcar-boxylic acid	61.510	C_7_H_6_O_5_	170.12	134.0500, 107.0377	Phenolic acids
4	Formylanthranilic acid	67.420	C_8_H_7_NO_3_	165.15	92.0499, 120.0445	Phenolic acids
5	Telmisartan	107.033	C_33_H_30_N_4_O_2_	514.63	276.2981	Phenolic acids
6	4-Aminosalicylic acid	163.254	C_7_H_7_NO_3_	153.13	108.0235	Phenolic acids
7	2-Fluorobenzoic acid	172.283	C_7_H_5_FO_2_	140.10	93.0368	Phenolic acids
8	Lasalocid	200.558	C_34_H_53_NaO_8_	612.77	131.0722	Phenolic acids
9	Afzelin	84.989	C_21_H_20_O_10_	431.0944	285.0321	Flavonoids
10	Apigenin-7-O-neohesperidoside	207.151	C_28_H_32_O_14_	577.1501	269.0421	Flavonoids
11	Cyanidin-3-O-alpha-arabinoside	129.516	C_20_H_18_O_10_	417.0802	287.0112	Flavonoids
12	Hesperidin	181.863	C_28_H_34_O_15_	609.1797	301.0224	Flavonoids
13	Naringenin-7-O-glucoside	117.097	C_33_H_42_O_19_	433.1118	151.0023	Flavonoids
14	Scutellarin	278.186	C_21_H_18_O_12_	461.0705	285.0404	Flavonoids

**Table 4:** The gradient elution procedure.

Elution Time (min)	Mobile Phase B Concentration (%)
0–0.5	95
0.5–7	linearly from 95 to 65
7–8	linearly from 65 to 40
8–9	maintained at 40
9–9.1	linearly from 40 to 95
9.1–12	maintained at 95

## Data Availability

The data presented in this study are available on request from the corresponding author.

## Supplementary Materials

The following supporting information can be downloaded at: https://www.mdpi.com/article/10.3390/molecules27082420/s1, Table S1: Information on all metabolites.
